# Predicting preoperative lymph node status in patients with cervical cancer: development of interpretable machine learning model and support for the biological plausibility

**DOI:** 10.3389/fimmu.2025.1654332

**Published:** 2025-10-10

**Authors:** Hui Shen, Yuting Jiang, Lihe Zhang, Qiao Zheng, Han Bai, Lihong Wu, Liu Du, Hongning Xie

**Affiliations:** Department of Ultrasonic Medicine, Fetal Medical Centre, the First Affiliated Hospital of Sun Yat-Sen University, Guangzhou, Guangdong, China

**Keywords:** cervical cancer, lymph node metastasis, monocyte, machine learning, SHAP value

## Abstract

**Background:**

Lymph node metastasis serves as a crucial prognostic risk factor for patients with cervical cancer. Accurate prediction of lymph node metastasis is important in guiding treatment selection. Therefore, our primary objective is the development and validation of machine learning models for predicting lymph node metastasis; the secondary objective is to utilize the sequencing data to provide biological plausibility.

**Methods:**

This study retrospectively included 292 cervical cancer patients and prospectively recruited 54 cervical cancer patients. Univariate and multivariate analysis were conducted to explore the risk factors associated with lymph node metastasis. Subsequently, cellular-level validation was performed using single cell RNA-sequencing data. The prognostic value of the risk factor was assessed through bulk RNA-sequencing analysis. Finally, patients were divided into train and retrospective test sets in a 7:3 ratio to develop five machine learning models, while using the prospective test set to validate the models. Additionally, the Shapley Additive Explanation method was employed to enhance the interpretability of the models’ decision processes.

**Results:**

Federation of Gynecology and Obstetrics stage (2018), squamous cell carcinoma antigen, monocyte count and platelet count were found to be significantly correlated with lymph node metastasis. Meanwhile, monocyte count was a significant risk factor (OR=2.28, *p* < 0.05). Single cell RNA-sequencing analysis revealed an increase in monocytes at IIIC1 stage compared to IB and IIB stages. Monocytes were significantly associated with prognosis and lymph node metastasis in the bulk RNA-sequencing. Finally, we developed and validated five machine learning models for predicting lymph node metastasis. The NNET model stood for its ability to predict lymph node metastasis (train set AUC: 0.86; retrospective test set AUC: 0.79; prospective test set: 0.76). In the interpretability of machine learning models, Shapley Additive Explanation values demonstrated the concrete contribution of each feature within the NNET model.

**Conclusions:**

This study investigated the notable association between monocyte count and lymph node metastasis, highlighting the importance of monocytes in cervical cancer via bulk RNA-sequencing and single cell RNA-sequencing analysis. The developed interpretable machine learning models effectively aid clinicians in decision-making processes. Additionally, the Shapley Additive Explanation method improved the applicability of these machine learning models in real world.

## 1 Introduction

Cervical cancer (CC) ranks as the fourth most prevalent cancer among women worldwide (1). In China, CC stands as a prevalent malignancy within the female reproductive system, ranking sixth in terms of incidence and seventh in mortality (2). According to some studies, the 5-year survival rate for patients diagnosed with early-stage CC without lymph node metastasis (LNM) ranges between 85-90%, contrasting with rates of only 50-55% for patients with LNM (3, 4). Therefore, LNM is one of the most important prognostic factors in patients with CC (5). Additionally, according to the 2018 International Federation of Gynecology and Obstetrics (FIGO) (2018) staging system for CC, patients with LNM were classified as IIIC stage and required concurrent chemoradiotherapy (CCRT), regardless of tumor size or parametrial infiltration (6, 7). Therefore, accurate diagnosis of LNM is crucial for improving prognosis and reducing mortality.

Traditionally, magnetic resonance imaging (MRI) and computed tomography (CT) are employed as diagnostic tools in the evaluation of CC (8). CT or MRI primarily identified LNM based on lymph node size; nevertheless, their sensitivity is limited, ranging only from 38% to 56% (9). Positron emission tomography/computed tomography (PET/CT) scan is more sensitive than CT or MRI alone; but the cost is relatively high and radiation exposure occurs (10). Currently, research on predicting LNM in CC primarily involves constructing radiomics models using medical imaging (11, 12). However, the construction of radiomics models requires manual delineation of regions of interest to extract radiomic features, resulting in poor reproducibility and posing challenges for real-world clinical applications (13).

In recent years, a growing body of research has revealed the correlation between chronic systemic inflammatory response and the progression and prognosis of tumors (14, 15). Peripheral blood parameters have been demonstrated to be associated with systemic inflammatory responses, and some peripheral blood parameters such as monocyte count (MO#), lymphocyte count (LY#), and lymphocyte monocyte ratio (LMR) have been found to be associated with cancer prognosis (16–18). In comparison to medical imaging, clinical features and peripheral blood parameters are more readily accessible in clinical practice and are cost-effective. Hence, peripheral blood parameters may provide new pathways for predicting LNM.

The primary aim of this study is to develop and validate various machine learning (ML) models using peripheral blood parameters to achieve accurate prediction of LNM risk in CC patients. The secondary objective is to provide biological plausibility for the peripheral blood parameters using single-cell RNA sequencing (scRNA-seq) data and bulk-RNA-sequencing (bulk-RNA-seq) data. Furthermore, the utilization of Shapley Additive Explanation (SHAP) values, an interpretable artificial intelligence (AI) technique, to explain the features in the models.

## 2 Methods

### 2.1 Clinical database

The data of all CC patients were obtained from the First Affiliated Hospital of Sun Yat-sen University. The retrospective dataset was built between January 2020 and December 2024. The prospective validation dataset was constructed between January 2025 and June 2025.The study adhered to the Helsinki ethical statement standards and was approved by the Ethics Review Committee of the First Affiliated Hospital of Sun Yat-sen University [approval number: (2023)141]. All participants agreed to the study and signed the informed consent forms.

The inclusion criteria were as follows: (1) patients aged ≥ 18 years; (2) patients who underwent radical hysterectomy with pelvic lymphadenectomy with pathologically confirmed CC. Exclusion criteria were as follows: (1) MRI and/or CT and/or PET/CT reveal LNM in the patient; (2) patients with combined other malignancies; (3) The clinical data is incomplete; (4) neuroendocrine carcinoma and other rare pathological types. The inclusion and exclusion criteria for cases are illustrated in **Supplementary Figure 1**.

### 2.2 Single cell RNA-sequencing database

ScRNA-seq data (GSE171894) were obtained from the GEO website (https://www.ncbi.nlm.nih.gov/geo/). Three samples were chosen, corresponding to FIGO IB, IIB, and IIIC1stages, respectively.

### 2.3 TCGA database

The TCGA data portal (https://portal.gdc.cancer.gov/) was used to obtain RNA gene expression data and corresponding clinical information for cervical squamous cell carcinoma and endocervical adenocarcinoma patients. We matched 304 samples retrieved from the TCGA database with their corresponding clinical data, ensuring that the samples had an overall survival (OS) period of more than 0 days, complete clinical stage, and age information. Ultimately, 235 samples were included for analysis.

### 2.4 Clinical data collection

The clinical data, lymph node status, and preoperative hematological information of all patients were retrospectively collected. Clinical information included age, FIGO (2018) stage, menstrual history and history of neoadjuvant chemotherapy (Neo-chemotherapy). The hematological data were collected, and included carbohydrate antigen 125 (CA125), carbohydrate antigen 19-9 (CA19-9), squamous cell carcinoma antigen (SCCA), neutrophil percentage (NEUT%), lymphocyte percentage (LY%), monocyte percentage (MO%), neutrophil count (NEUT#), LY#, MO# and platelet count (PLT#). Furthermore, inflammation-related indicators were calculated, and included the neutrophil lymphocyte ratio (NLR), LMR, neutrophil platelet ratio (NPR), the systemic immune-inflammation index (SII; SII=PLT# **×** NEUT#/LY#), systemic inflammatory response Index (SIRI; SIRI=NEUT# **×** MO#/LY#) and pan-immune-inflammation value (PIV; PIV=NEUT# **×** PLT# **×** MO#/LY#). The receiver operating characteristic (ROC) curve was constructed to determine the cut-off values of the hematological data for predicting the presence of LNM.

### 2.5 ScRNA-seq and bulk-RNA-seq data processing

The Seurat R package (version 4.4.0) was employed to analyze the scRNA-seq data. Standard scRNA-seq filtering excludes low-quality cells with less than 200 or over 7, 500 expressed genes, or unique molecular identifiers (UMIs) originating from the mitochondrial genome exceeding 20%, or UMIs from the erythrocyte genome surpassing 5%. Cells were normalized and scaled with the default parameters and their highly variable features were determined using FindVariableFeatures function. PCA analysis was then performed with the identified variable features. Dimension reduction and clustering were conducted using FindNeighbors and FindClusters functions, respectively. Finally, Uniform Manifold Approximation and Projection (UMAP) were performed for visualization. Cell types were annotated to known biological types with canonical marker genes. Based on the top differentially expressed genes (DEGs) of each cell type in scRNA-seq, single sample gene set enrichment analysis (ssGSEA) was performed for all cell types in the bulk-RNA-seq data.

The CIBERSORT R package was used to investigate the proportions of immune cells in diverse TCGA samples, and cox regression was utilized to evaluate the prognostic significance of distinct immune cell types for CC patients. Furthermore, we also compared the differential expression of monocyte-related genes between samples with and without LNM.

### 2.6 Feature selection

To address the issue of multicollinearity among variables in the study, we utilized Variance Inflation Factor (VIF) to assess the various clinical variables. We employed the method of feature elimination with cross‐validation (RFECV) for feature selection. RFECV iteratively eliminates features considered least important and employs cross-validation to assess the performance of the selected feature subsets at each iteration, thereby determining the optimal number of features. The key benefit of RFECV lies in mitigating the subjectivity associated with feature selection and improving the accuracy and generalization ability of the model.

### 2.7 Model development and evaluation

We constructed and tested five ML models: logistic regression (LR), random forest (RF), naive bayes (NB), decision tree (DT), and neural network (NNET). The patients were separated into a train set and a retrospective test set (ratio 7: 3) and performed the tenfold cross‐validation to train models. In the train, retrospective test and prospective test sets, the area under the receiver operating characteristic curve (AUC), accuracy, sensitivity, specificity and Brier score, were estimated. Utilizing the De-long test to compare whether there is the significant difference in AUC among the various ML models. Compare the improvement in predictive effect between the ML models using the Net Reclassification Index (NRI) and the integrated discrimination improvement (IDI). Calibration curves were utilized to illustrate the correspondence between the predicted probabilities and the actual outcomes. Decision curve analysis (DCA) was utilized to assess the net benefit of the models.

### 2.8 Interpretability analysis of model and web-based application

To mitigate the mistrust associated with ML algorithms due to their “black box” nature, we applied SHAP values to interpret our ML models. SHAP theory, which is rooted in cooperative game theory, offers a robust and highly interpretable framework that quantifies the specific influence and relative importance of each feature on the model’s predictive outcomes.

The optimal predictive model was deployed on the ShinyApps website (https://www.shinyapps.io/), where we established an accessible online computational platform. This web-based application enables real-time LNM prediction for CC patients, thus facilitating the application of the model in the real world.

### 2.9 Statistical analysis

Categorical variables were represented as frequencies and percentages. The comparison of categorical data between groups was conducted using the χ² test or Fisher’s exact test. We used univariate and multivariate logistic regression analysis to identify risk factors and calculate their odd ratios (ORs) and 95% confidence intervals (CIs). Utilizing the R package “caret” to construct various ML models. All statistical analysis was performed with R, version 4.2.2 software (R Project for Statistical Computing). A two-tailed *p*-value < 0.05 was considered statistically significant.

## 3 Results

### 3.1 Baseline characteristics

We retrospective reviewed 333 cases of patients with CC who underwent radical hysterectomy and pelvic lymphadenectomy. Among them, 21 patients were diagnosed with LNM on preoperative imaging studies, 17 patients had neuroendocrine carcinoma and other rare pathological types, 2 patients had concomitant other malignant tumors, and one patient had missing preoperative clinical data, all of whom were excluded. Ultimately, 292 patients met the eligibility criteria and were included in the train set (n=204) and the retrospective test set (n=88). We prospectively recruited 64 CC patients, where 6 individuals were identified with LNM through preoperative imaging studies, and 4 patients exhibited neuroendocrine carcinoma and other rare pathological types. Ultimately, 54 CC patients were selected as the prospective test set. The characteristics of the train, retrospective test and prospective test sets are shown in **Table 1**. The incidence of LNM in the train set, retrospective test and prospective test sets is 23.53%, 17.05% and 11.11%, respectively (*p*=0.093). The distribution of age, FIGO (2018) stage, and other characteristics showed no significant differences among different datasets.

**Table 1:** Characteristics of the train and test sets.

Variables	Train set (N=204)	Retrospective test set (N=88)	Prospective test set (N=54)	*P* value
Age, N (%):				0.441
≤35 years	16 (7.84%)	11 (12.50%)	5 (9.26%)	
>35 years	188 (92.16%)	77 (87.50%)	49 (90.74%)	
Menstrual history, N (%):				0.475
No	124 (60.78%)	48 (54.55%)	29 (53.70%)	
Yes	80 (39.22%)	40 (45.45%)	25 (46.30%)	
Neo-treatment, N (%):				0.056
No	172 (84.31%)	70 (79.55%)	51 (94.44%)	
Yes	32 (15.69%)	18 (20.45%)	3 (5.56%)	
FIGO (2018), n (%)				
IB1	27(13.24%)	9(10.23%)	8(14.81%)	0.783
IB2	71(34.80%)	31(35.23%)	23(42.59%)	
IB3	19(9.31%)	11(12.50%)	7(12.96%)	
IIA1	49(24.02%)	18(20.45%)	9(16.67%)	
IIA2	30(14.71%)	13(14.77%)	4(7.41%)	
IIB	8(3.92%)	6(6.82%)	3(5.56%)	
Type, N (%):				0.072
Adenocarcinoma	39 (19.12%)	11 (12.50%)	17 (31.48%)	
Squamous cell carcinoma	159 (77.94%)	73 (82.95%)	35 (64.81%)	
Adenosquamous carcinoma	6 (2.94%)	4 (4.55%)	2 (3.70%)	
CA125, N (%):				0.194
≤35 U/mL	184 (90.20%)	83 (94.32%)	46 (85.19%)	
>35 U/mL	20 (9.80%)	5 (5.68%)	8 (14.81%)	
CA199, N (%):				0.324
≤35 U/mL	189 (92.65%)	84 (95.45%)	48 (88.89%)	
>35 U/mL	15 (7.35%)	4 (4.55%)	6 (11.11%)	
SCCA, N (%):				0.649
≤1.5 ug/L	117 (57.35%)	46 (52.27%)	32 (59.26%)	
>1.5 ug/L	87 (42.65%)	42 (47.73%)	22 (40.74%)	
NEUT%, N (%):				0.501
≤0.56	67 (32.84%)	35 (39.77%)	20 (37.04%)	
>0.56	137 (67.16%)	53 (60.23%)	34 (62.96%)	
LY%, N (%):				0.979
≤0.33	130 (63.73%)	55 (62.50%)	34 (62.96%)	
>0.33	74 (36.27%)	33 (37.50%)	20 (37.04%)	
MO%, N (%):				0.852
≤0.06	45 (22.06%)	19 (21.59%)	10 (18.52%)	
>0.06	159 (77.94%)	69 (78.41%)	44 (81.48%)	
NEUT#, N (%):				0.291
≤3.61×10^9/L	91 (44.61%)	48 (54.55%)	25 (46.30%)	
>3.61×10^9/L	113 (55.39%)	40 (45.45%)	29 (53.70%)	
LY#, N (%):				0.888
≤1.95×10^9/L	110 (53.92%)	49 (55.68%)	31 (57.41%)	
>1.95×10^9/L	94 (46.08%)	39 (44.32%)	23 (42.59%)	
MO#, N (%):				0.395
≤0.46×10^9/L	90 (44.12%)	46 (52.27%)	27 (50.00%)	
>0.46×10^9/L	114 (55.88%)	42 (47.73%)	27 (50.00%)	
PLT, N (%):				0.413
≤258.50×10^9/L	117 (57.35%)	54 (61.36%)	27 (50.00%)	
>258.50×10^9/L	87 (42.65%)	34 (38.64%)	27 (50.00%)	
NLR, N (%):				0.807
≤2.27	140 (68.63%)	57 (64.77%)	36 (66.67%)	
>2.27	64 (31.37%)	31 (35.23%)	18 (33.33%)	
LMR, N (%):				0.143
≤6.23	178 (87.25%)	83 (94.32%)	50 (92.59%)	
>6.23	26 (12.75%)	5 (5.68%)	4 (7.41%)	
NPR, N (%):				0.899
≤14.86	103 (50.49%)	44 (50.00%)	29 (53.70%)	
>14.86	101 (49.51%)	44 (50.00%)	25 (46.30%)	
SIRI, N (%):				0.459
≤0.62	55 (26.96%)	18 (20.45%)	15 (27.78%)	
>0.62	149 (73.04%)	70 (79.55%)	39 (72.22%)	
SII, N (%):				0.918
≤506.65	111 (54.41%)	46 (52.27%)	30 (55.56%)	
>506.65	93 (45.59%)	42 (47.73%)	24 (44.44%)	
PIV, N (%):				0.775
≤400.36	162 (79.41%)	73 (82.95%)	43 (79.63%)	
>400.36	42 (20.59%)	15 (17.05%)	11 (20.37%)	
LNM, N (%):				0.093
No	156 (76.47%)	73 (82.95%)	48 (88.89%)	
Yes	48 (23.53%)	15 (17.05%)	6 (11.11%)	

### 3.2 Univariate and multivariate analysis for LNM

We investigated the independent risk factors for LNM among all patients with CC (**Table 2**). The univariate analysis indicated that FIGO (2018) stage, SCCA, MO%, MO#, PLT# and LMR were all linked to LNM (*p* < 0.05). Meanwhile, the multivariate analysis validated FIGO (2018) stage, SCCA, MO# and PLT# as independent factors associated with LNM (*p* < 0.05). Within the hematological data, MO# emerged as an independent risk factor for predicting LNM, boasting the highest OR value (OR=2.28).

**Table 2:** Univariate and multivariate logistic regression analysis of LNM.

Variables	Univariate analysis		Multivariate analysis	
	OR (95%CI)	*P* value	OR (95%CI)	*P* value
Age		0.078		
≤35 years	Ref			
>35 years	3.74(1.07-23.64)			
Menstrual history		0.797		
No	Ref			
Yes	0.93(0.52-1.63)			
Neo-treatment		0.766		
No	Ref			
Yes	0.89(0.40-1.84)			
FIGO (2018)				
IB1	Ref		Ref	
IB2	2.70(0.71-17.80)	0.204	2.02(0.50-13.71)	0.381
IB3	9.84(2.34-68.08)	0.005	9.66(2.12-70.15)	0.008
IIA1	8.31(2.24-54.12)	0.006	6.93(1.73-46.89)	0.016
IIA2	6.58(1.63-44.48)	0.019	5.19(1.21-36.31)	0.047
IIB	2.83(0.31-25.84)	0.323	3.1(0.33-29.74)	0.297
Type				
Adenocarcinoma	Ref			
Squamous cell carcinoma	1.35(0.64-3.12)	0.454		
Adenosquamous carcinoma	0.51(0.03-3.12)	0.542		
CA125		0.190		
≤35 U/mL	Ref			
>35 U/mL	1.81(0.71-4.31)			
CA199		0.954		
≤35 U/mL	Ref			
>35 U/mL	0.97(0.27-2.78)			
SCCA		0.002		0.041
≤1.5 ug/L	Ref		Ref	
>1.5 ug/L	2.51(1.42-4.50)		1.97(1.03-3.81)	
NEUT%		0.235		
≤0.56	Ref			
>0.56	0.71(0.40-1.26)			
LY%		0.249		
≤0.33	Ref			
>0.33	1.4(0.79-2.46)			
MO%		0.010		0.425
≤0.06	Ref		Ref	
>0.06	3.22(1.42-8.70)		1.52(0.57-4.59)	
NEUT#		0.254		
≤3.61×10^9/L	Ref			
>3.61×10^9/L	0.72(0.41-1.26)			
LY#		0.181		
≤1.95×10^9/L	Ref			
>1.95×10^9/L	0.68(0.38-1.19)			
MO#		0.019		0.023
≤0.46×10^9/L	Ref		Ref	
>0.46×10^9/L	2.02(1.14-3.67)		2.28(1.14-4.71)	
PLT#		0.042		0.007
≤258.50×10^9/L	Ref		Ref	
>258.50×10^9/L	0.54(0.29-0.97)		0.39(0.19-76)	
NLR		0.290		
≤2.27	Ref			
>2.27	0.72(0.38-1.31)			
LMR		0.030		0.132
≤6.23	Ref		Ref	
>6.23	0.11(0.01-0.52)		0.19(0.01-1.13)	
NPR		0.181		
≤14.86	Ref			
>14.86	1.47(0.84-2.59)			
SIRI		0.063		
≤0.62	Ref			
>0.62	2.01(1.00-4.41)			
SII	0.65	0.145		
≤506.65	Ref			
>506.65	0.65(0.37-1.15)			
PIV		0.240		
≤400.36	Ref			
>400.36	0.63(0.27-1.31)			

Meanwhile, in order to quantify the potential additional value of MO#, we constructed two logistic regression models to predict LNM: model1(FIGO+SCCA+PLT#) and model2(FIGO+SCCA+PLT#+MO#). By comparing the AUC of the two models (model1 vs. model2=0.68 vs. 0.74; *p* < 0.05), we observed a significant enhancement in the predictive capability of model2 (**Supplementary Table 1**). Subsequently, our aim was to offer the potential biological plausibility of MO# through multi-omics analysis.

### 3.3 ScRNA-seq analysis: monocytes show an increase in CC patients with LNM

**Figure 1:**
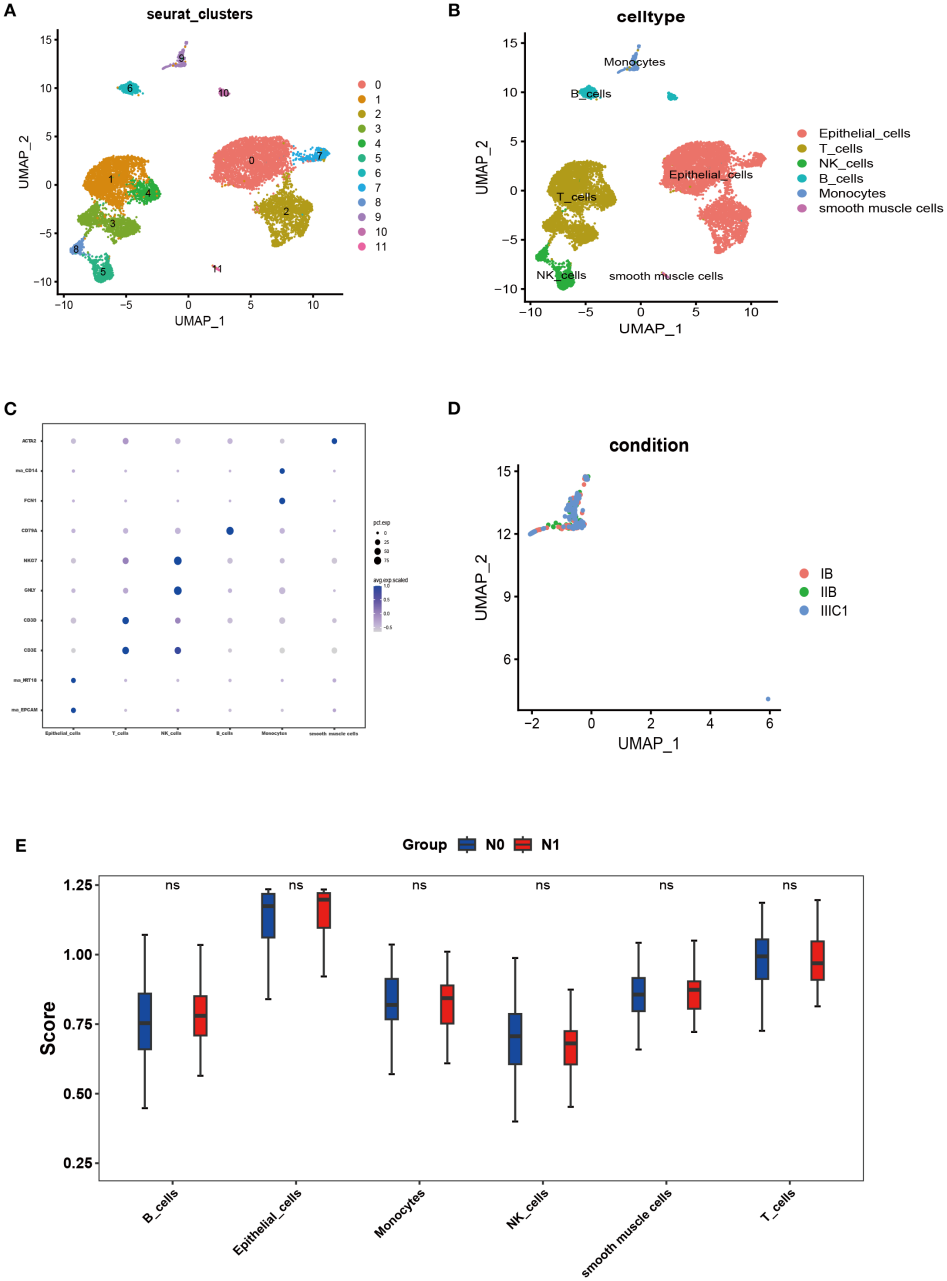
Single-cell landscape of CC. **(A, B)** The cells were clustered into 12 clusters and annotated into 6 kinds of cell types. **(C)** Heatmap showing expression levels of specific markers in each cell cluster. **(D)** UMAP plot colored by cell of FIGO stage: IB stage (red), IIB stage (green), and IIIC1 stage (bule). **(E)** SsGSEA analysis was performed for all cell types through the bulk-RNA-seq data.

The scRNA-seq analysis was conducted on three samples (FIGO IB/IIB/IIIC stage) derived from the scRNA-seq dataset GSE171894. A total of 11,011cells were obtained after stringent filtering. These cells were further classified into 12 different clusters (**Figure 1A**). The annotation results were derived from cell marker genes, and the heatmap displayed the marker genes (**Figures 1B, C**). In the **Figure 1C**, these 12 cell clusters were assigned to six different cell types, including epithelial cells (marked with *EPCAM* and *KRT18*), T cells (marked with *CD3D* and *CD3E*), NK cells ((marked with *GNLY* and *NKG7*), Monocytes (marked with *FCN1* and *CD14*), B cells (marked with *CD79A*) and smooth muscle cells (marked with *ACTA2*). **Figure 1D** illustrated an increase in monocytes in the sample corresponding to IIIC1 stage compared to those from IB and IIB stages. Meanwhile at the Bulk-RNA-seq level, there was a slight increase in the proportion of monocytes in patients with LNM; although it did not reach statistical significance (**Figure 1E**).

### 3.4 TCGA analysis: monocytes are a risk factor that influences the prognosis of CC patients

In the TCGA database, we utilized the R software CIBERSORT to calculate the proportions of 22 distinct immune cell types. Univariate and multivariate Cox regression analyses were conducted to explore the potential prognostic value of 22 immune cell subtypes and clinical features. In the **Table 3**, the results revealed monocytes and resting mast cells were significantly correlated with OS (*p* < 0.05). Meanwhile, considering that the Hazard Ratio (HR) for monocyte exceeded 1, it consequently emerged as a significant risk factor for prognosis.

**Table 3:** Univariate and multivariate Cox regression analysis for predicting OS.

Characteristics	Univariate analysis		Multivariate analysis	
	HR (95% CI)	*P* value	HR (95% CI)	*P* value
B.cells.naive	1.04 (0.79-1.38)	0.772		
B.cells.memory	0.68 (0.34-1.39)	0.293		
Plasma.cells	0.90 (0.65-1.24)	0.507		
T.cells.CD8	0.64 (0.42-0.98)	<0.05	0.78 (0.45-1.35)	0.379
T.cells.CD4.Naive	NA	NA		
T.cells.CD4.memory. resting	1.15 (0.85-1.57)	0.369		
T.cells.CD4.memory. activated	0.80 (0.54-1.16)	0.237		
T.cells.follicular.helper	0.82 (0.59-1.13)	0.226		
T.cells.regulatory..Tregs.	0.96 (0.71-1.29)	0.774		
T.cells.gamma.delta	0.58 (0.30-1.15)	0.121		
NK.cells.resting	0.85 (0.48-1.51)	0.575		
NK.cells.activated	0.70 (0.40-1.23)	0.219		
Monocytes	1.49 (1.09-2.05)	<0.05	1.72 (1.25-2.37)	<0.05
Macrophages.M0	1.61 (1.21-2.14)	<0.05	1.18 (0.74-1.90)	0.489
Macrophages.M1	0.94 (0.67-1.32)	0.737		
Macrophages.M2	1.39 (0.96-2.03)	0.085		
Dendritic.cells.resting	0.59 (0.36-0.96)	<0.05	0.75 (0.48-1.17)	0.203
Dendritic.cells.activated	1.35 (0.87-2.09)	0.179		
Mast.cells.resting	0.52 (0.34-0.81)	<0.05	0.59 (0.37-0.93)	<0.05
Mast.cells.activated	2.26 (1.54-3.31)	0.282		
Eosinophils	1.18 (0.75-1.85)	0.467		
Neutrophils	1.09 (0.79-1.51)	0.604		
Age	1.02 (0.99-1.04)	0.190		
Stage	1.55 (1.14-2.11)	<0.05	1.32 (0.95-1.83)	0.098

**Figure 2:**
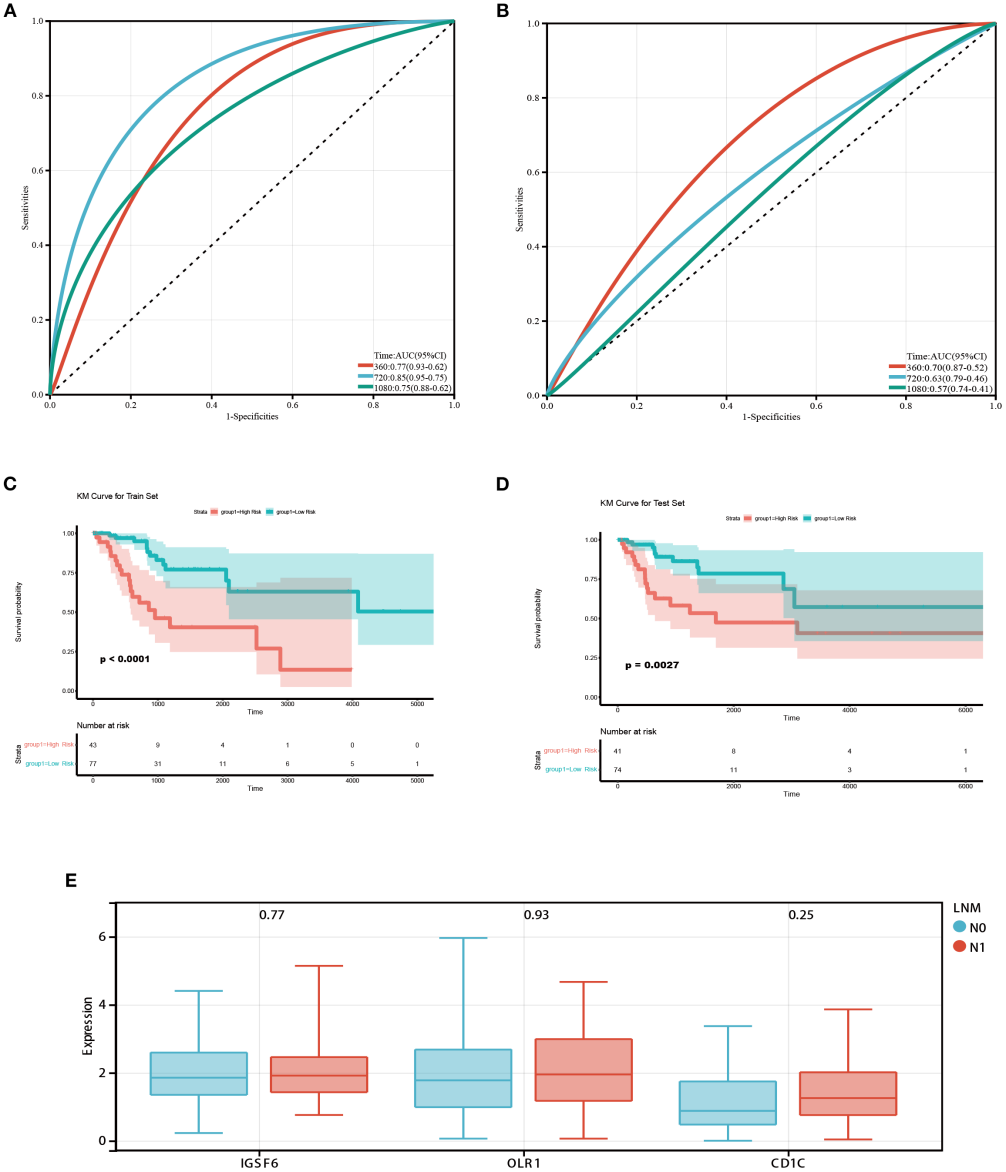
Prognostic value of monocytes in the TCGA database. **(A)** ROC curves of the prognostic model based on monocytes and resting mast cells in the training cohort. The AUC values at 1,2 and 3 years were 0.77, 0.85, and 0.75 respectively. **(B)** ROC curves of the prognostic model in the testing cohort. The AUC values at 1,2 and 3 years were 0.70, 0.63, and 0.57 respectively. (**C, D)** Survival analysis in training and testing cohort. Patients classified as high-risk exhibited shorter OS. **(E)** Box diagram showed the difference of monocyte-related genes expression between patients with and without LNM.

Subsequently, ROC curves were used to evaluate the prognostic capability of monocytes and resting mast cells. The TCGA dataset was divided into training and testing cohorts in a 5:5 ratio. In the training cohort, it was observed that the prognostic model demonstrated an AUC of 0.77, 0.85, and 0.75 at 1-, 2-, and 3-year intervals, respectively (**Figure 2A**). In the testing cohort, the prognostic model displayed an AUC of 0.70, 0.63, and 0.57 at 1-, 2-, and 3-year intervals, respectively (**Figure 2B**). Additionally, patients were categorized into high/low risk groups according to their risk scores, and subsequently underwent Kaplan-Meier survival analysis. In both the training and testing cohorts, we observed that patients classified as high-risk exhibited shorter OS (**Figures 2C, D**). In addition, we further explored the differences in the expression of significantly expressed genes of monocytes (IGSF6, OLR1 and CD1C) between patients with or without LNM. We found that the expressions of IGSF6, OLR1 and CD1C increased in CC patients with LNM (**Figure 2E**).

### 3.5 Feature selection

When multicollinearity is present among variables, it can result in instability in regression outcomes, thereby diminishing predictive capability. In the **Supplementary Table 2**, we calculated the VIF among the variables and found no significant signs of multicollinearity (VIF ≤ 3.62). Next, we employed RFECV strategy to determine the optimal feature subset for each ML model. This method utilized ten‐fold cross‐validation based on five ML classifiers, using the accuracy as the evaluation criterion to automatically select the optimal number of features. **Supplementary Figures 2–3** presented the results of the RFECV method for feature selection.

### 3.6 Prediction performance of different ML models

**Figure 3:**
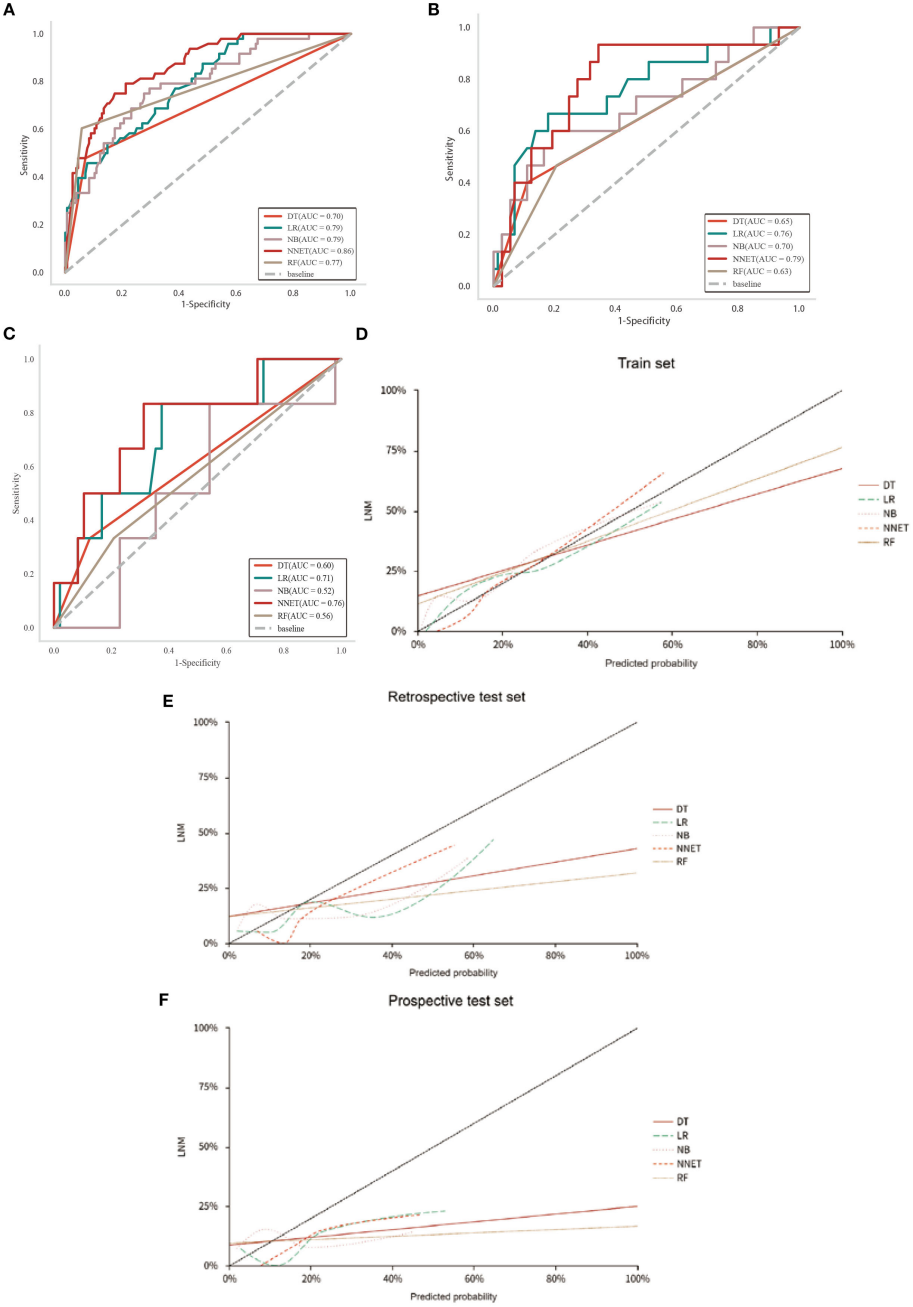
Performance of the five ML models in predicting LNM in patients with CC. **(A-C)**: ROC curves of train, retrospective test and prospective test sets. **(D-F)**: Calibration curves for five ML models across train, retrospective test and prospective test sets.

To ensure the stability and reliability of our ML models, ten-fold cross-validation was conducted on the training set for tuning, ultimately generating the optimal model. Of the ML models used to predict LNM in the train set, NNET model exhibited the highest AUC (0.86, 95% CI: 0.81-0.92), followed by LR model with an AUC of 0.79 (**Figure 3A**; **Table 4**). In the retrospective test set and prospective test set, the NNET model also achieved a higher AUC value of 0.79/0.76 (**Figures 3B, C**; **Table 4**). Meanwhile, the results of the De Long test indicated that the AUC of the NNET model demonstrated a statistically significant difference compared to all other models in the train set (*p* < 0.05) (**Supplementary Table 6**). Next, in terms of calibration, the NNET model also exhibited superior performance when comparing the calibration curves and Brier scores (**Figures 3D-F**; **Supplementary Table 4**). Furthermore, through the comparison of the NRI and IDI between the NNET model and the four other ML models, we found that the reclassification and discriminatory ability of the NNET model improved across the train set, retrospective test and prospective test set (**Supplementary Table 7**).

**Table 4:** Prediction efficacy of five ML models in train and test sets.

Train set				
Model	AUC (95% CI)	Sensitivity (95% CI)	Specificity (95% CI)	Accuracy (95% CI)
NB	0.79 (0.72-0.86)	0.77 (0.65-0.89)	0.71 (0.63-0.78)	0.72 (0.66-0.78)
DT	0.70 (0.63-0.78)	0.48 (0.34-0.62)	0.93 (0.89-0.97)	0.82 (0.77-0.88)
RF	0.77 (0.70-0.85)	0.60 (0.47-0.74)	0.94 (0.91-0.98)	0.83 (0.79-0.89)
NNET	0.86 (0.81-0.92)	0.79 (0.68-0.91)	0.79 (0.72-0.85)	0.79 (0.73-0.85)
LR	0.79 (0.72-0.86)	0.71 (0.58-0.84)	0.71 (0.63-0.78)	0.71 (0.64-0.77)
Retrospective test Set				
Model	AUC (95% CI)	Sensitivity (95% CI)	Specificity (95% CI)	Accuracy (95% CI)
NB	0.70 (0.53-0.86)	0.60 (0.35-0.85)	0.81 (0.71-0.90)	0.77 (0.69-0.86)
DT	0.65 (0.51-0.78)	0.40 (0.15-0.65)	0.89 (0.82-0.96)	0.81 (0.72-0.89)
RF	0.63 (0.49-0.77)	0.47 (0.21-0.72)	0.79 (0.70-0.89)	0.78 (0.70-0.87)
NNET	0.79 (0.67-0.92)	0.93 (0.81-1.00)	0.66 (0.55-0.77)	0.70 (0.61-0.80)
LR	0.76 (0.61-0.91)	0.67 (0.43-0.91)	0.82 (0.73-0.91)	0.80 (0.71-0.88)
Prospective test set				
Model	AUC (95% CI)	Sensitivity (95% CI)	Specificity (95% CI)	Accuracy (95% CI)
NB	0.52 (0.28-0.77)	0.83 (0.54-1.00)	0.46 (0.32-0.60)	0.50 (0.37-0.63)
DT	0.60 (0.39-0.82)	0.33 (0.04-0.71)	0.88 (0.78-0.97)	0.81 (0.71-0.92)
RF	0.56 (0.35-0.78)	0.33 (0.04-0.71)	0.79 (0.68-0.91)	0.74 (0.62-0.86)
NNET	0.76 (0.54-0.98)	0.83 (0.54-1.00)	0.69 (0.56-0.82)	0.70 (0.58-0.83)
LR	0.71 (0.49- 0.93)	0.83 (0.54-1.00)	0.63 (0.49-0.76)	0.65 (0.52-0.78)

### 3.7 Interpretability analysis based on SHAP

**Figure 4:**
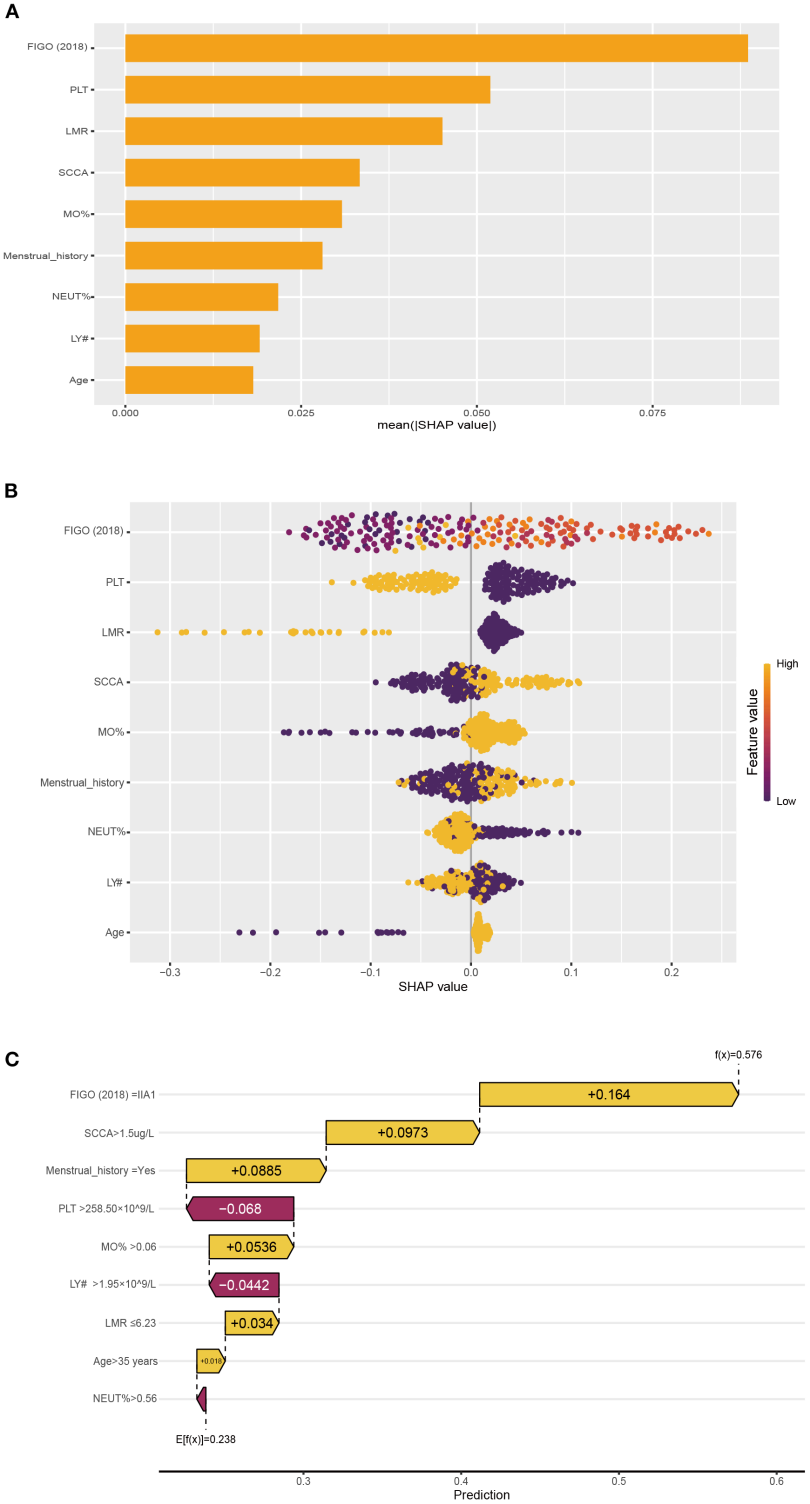
The NNET model's interpretation. **(A)**: SHAP value ranking of the variables in the model. **(B)**: SHAP honeycomb diagram of the NNET model. **(C)**: The interpretation of the NNET model prediction result for a single sample.

SHAP values indicate the contributions of individual variables to the predictive classification model results, aiding in interpreting the influence and importance of each feature in the model’s decision-making process. Therefore, we calculated SHAP value of NNET model to interpret and visualize prediction results. **Figure 4A** illustrated a bar graph displaying feature importance scores derived from SHAP values. This visualization demonstrated that the FIGO (2018) stage exerted the most significant influence on the model predictions, followed by PLT, LMR, SCCA and MO%. At the same time, in the **Figure 4B**, each point on the graph represents the SHAP value for an individual sample, where points closer to purple indicate higher values, whereas those closer to yellow signify lower values. So, the **Figure 4B** visually illustrated the direction and strength of the influence of each feature on the model prediction. Notably, advanced FIGO (2018) stage, high SCCA level, high MO% level, and increased age significantly elevated the risk of LNM. In addition, one of the 292 patients in our database were selected randomly for result exhibition (**Figure 4C**). According to the algorithm, the specific value of each feature in the NNET model is transformed into a probability and superimposed to form the overall probability of LNM. Based on the model prediction, the probability of LNM for this patient was estimated to be 0.576. **Supplementary Figure 5** illustrated the impact of these top 5 variables on the NNET model predictions.

### 3.8 Online web assessment tool for LNM in CC

**Figure 5:**
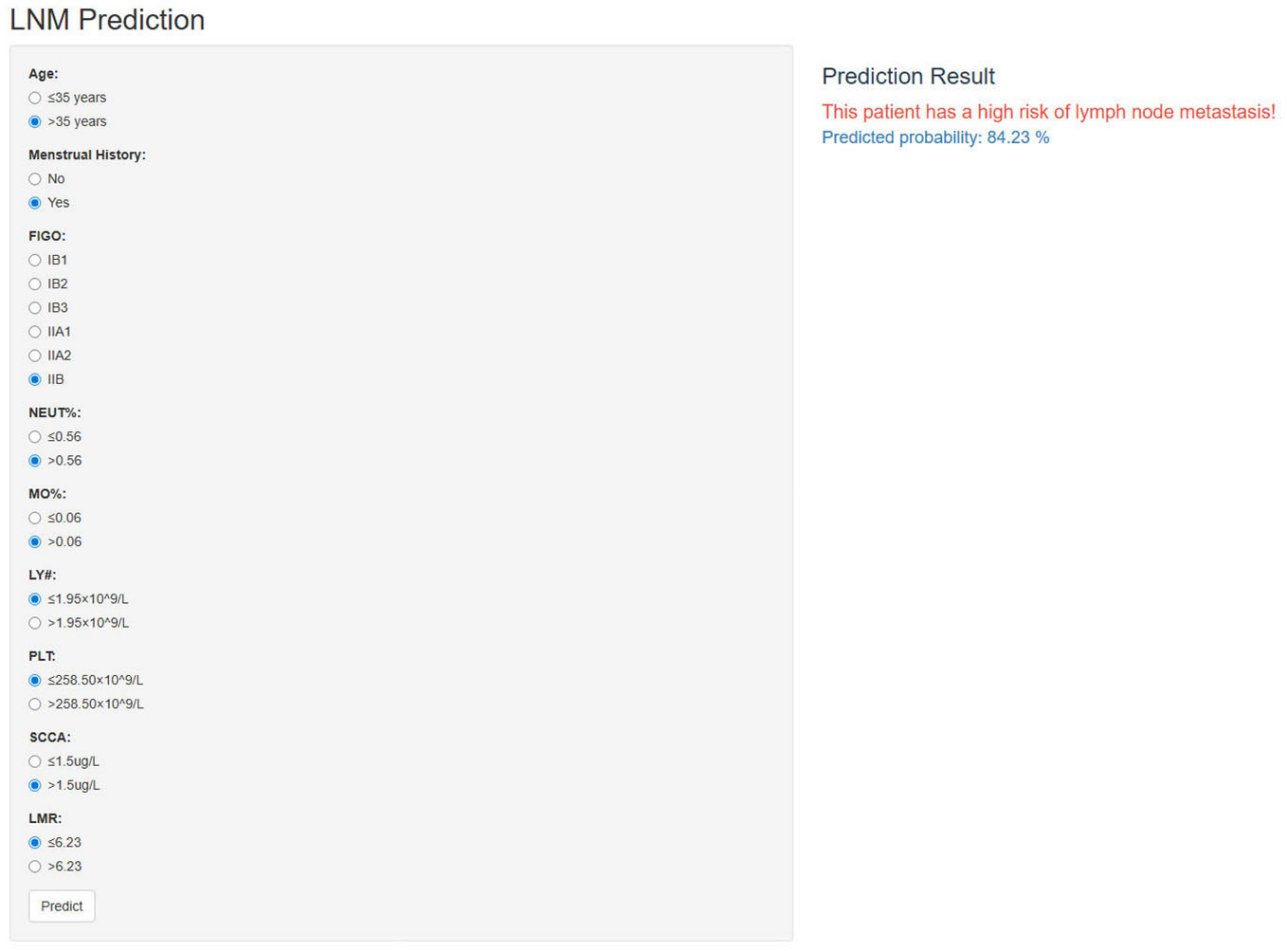
The online web-based application for predicting LNM in CC.

The incorporation of the NNET model into a publicly accessible web-based calculator (https://cclnmpredictor.shinyapps.io/shinyapp/) enabled clinicians to evaluate the risk of LNM in real-time (**Figure 5**).

## 4 Discussion

In this study, we have identified FIGO (2018) stage, SCCA, MO#, and PLT# as significant variables for predicting LNM in CC through univariate and multivariate analysis. Meanwhile, scRNA-seq analysis revealed an increased population of monocytes in IIIC1 stage compared to IB and IIB stages. In the bulk RNA-seq, monocytes showed significant correlation with LNM and the prognosis of CC. Moreover, a survival prediction model constructed based on monocytes and resting mast cells demonstrated moderate predictive accuracy, and Individuals at low-risk exhibit extended OS. Lastly, we have developed and validated five ML models for predicting LNM. Research indicated that the NNET model displayed excellent performance in predicting LNM metastasis (train set AUC: 0.86; retrospective test set AUC: 0.79; prospective test set: 0.76). The ML model could assist clinicians in adjusting the clinical staging of radiologically negative patients, thereby guiding clinical decisions, such as determining the necessity for additional neoadjuvant therapy.

Chronic inflammation is intricately linked to the initiation, proliferation, invasion, metastasis, and apoptosis (18). With the advancement of research, an increasing number of studies validated that the prognosis of cancer patients hinged not only on tumor-related factors but also on the systemic inflammatory response of the individuals (19). Peripheral blood cells reflect the inflammatory status of patients, and numerous studies have showed that peripheral blood monocytes serve as independent prognostic factors for various cancer patients (20–22). Our study also has revealed that peripheral blood monocytes are significant risk factors in LNM (OR=2.28). Simultaneously, monocytes are also associated with the bad prognosis of CC. In addition, immune cells infiltrating within tumor tissue are extravasated from peripheral blood (23). The results of our scRNA-seq also mirror these findings, with an increase in the number of monocytes observed in IIIC1 stage samples compared to IB/IIB stage samples, corroborating our clinical dataset.

Peripheral blood monocytes play an important role in tumors, yet the mechanisms underlying their involvement remain unclear. Currently, the prevailing hypothesis posits a close association between peripheral blood monocytes and tumor-associated macrophages (TAMs) within the tumor microenvironment. CD14^+^CD16^+^ monocytes exhibit Tie 2 expression, representing an angiopoietin receptor (Tie 2/Tek) present in the human peripheral blood monocytes with notable tumor-promoting and proangiogenic properties (24). Ang 2, a ligand of Tie 2, is primarily identified within cancer cells, and may induce transmigration of Tie2/CD14^+^CD16^+^ monocytes into the tumor tissues (25, 26). Following recruitment to the tumor microenvironment from the peripheral blood, monocytes undergo differentiation into TAMs under the influence of cytokines and chemokines produced by tumor cells (27). TAM, originating from peripheral blood monocytes, possess angiogenic characteristics that promote tumor growth and metastasis, alongside participating in the inhibition of anti-tumor immune responses (20, 28).

The LNM prediction model introduced in this study showed exceptional effectiveness and exhibited promising clinical applicability. A meta-analysis of 23 studies unveiled that AI models developed using medical images achieved an AUC of 0.76, contrasting with radiologists who achieved a lower AUC of 0.65 (29). Meanwhile, The ML model constructed using MRI radiomic features and clinical characteristics obtained an AUC of 0.745 (30). In contrast to radiomics-based models and radiologists, the ML model we constructed using clinical features and hematological data exhibits superior efficacy (AUC=0.79). Moreover, the model’s notable clinical applicability arises from its dependence on easily accessible patient data like FIGO (2018) stage and hematological data, making it readily applicable in real world. Our model serves as a fundamental tool for clinicians to make personalized clinical decisions. According to clinical guidelines, CC patients identified with LNM during preoperative assessment are categorized as IIIC stage, for which CCRT is the standard treatment over radical hysterectomy. Accurate preoperative assessment of lymph node status will significantly minimize unnecessary interventions for CC patients and optimize treatment selection.

In contrast to prior studies primarily focused on predictive performance, our research employed SHAP values to enhance the interpretability of model predictions. With the continual advancement of science and technology, AI has been extensively implemented in the field of healthcare (31). Nevertheless, this transformation has also ushered in certain challenges, given that AI models operate as black boxes, rendering the interpretability of their prediction processes nearly inscrutable (32). In our study, we employed SHAP values to enhance the interpretability of ML models. The SHAP method utilizes game-theoretic techniques to assign significance to individual input features, facilitating a more profound understanding of model behavior (33). In general, SHAP values guarantee the accuracy and interpretability of our ML model, making it appropriate for practical clinical implementation.

Our study had some limitations. Firstly, the analysis was conducted at a single center, and larger external validation cohort is imperatively warranted. Secondly, we elaborated on the significance of monocytes by using publicly available bulk RNA-seq and scRNA-seq data. However, only three samples were used to perform the scRNA-seq analysis, which is insufficient to support the observation of increased monocytes in the IIIC1 stage. To strengthen our findings, additional sequencing data related to CC will be necessary to provide more robust evidence. Next, the impact of spectral bias has not been adequately considered when developing the ML models; therefore, future validation across diverse patient populations is essential to enhance its clinical applicability. Furthermore, our study did not extensively investigate the relationship between peripheral blood monocytes and monocytes within tumor tissue. Finally, hematological data associated with CC, including carcinoembryonic antigen and Human epididymis protein 4, were excluded from the analysis due to substantial missing data.

## 5 Conclusions

This study demonstrated a significant association between MO# and LNM in CC, assessing the potential value of monocytes in CC through a comprehensive evaluation using bulk RNA-seq and scRNA-seq. Meanwhile, by incorporating clinical characteristics and hematological data, five ML models were constructed to predict LNM, with the NNET model exhibiting the strongest predictive performance, offering decision support for clinicians. Additionally, the SHAP method was utilized to elucidate the decision-making process of the ML models, thereby enhancing their applicability in real world.

## Data Availability

The original contributions presented in the study are included in the article/**Supplementary Material**. Further inquiries can be directed to the corresponding authors.

## Glossary

CC: Cervical cancer
LNM: Lymph node metastasis
FIGO: International Federation of Gynecology and Obstetrics
CCRT: Concurrent chemoradiotherapy
MRI: Magnetic resonance imaging
CT: Computed tomography
PET/CT: Positron emission tomography/computed tomography
MO#: Monocyte count
LY#: Lymphocyte count
LMR: Lymphocyte monocyte ratio
ML: Machine learning
SHAP: Shapley Additive Explanation
scRNA-seq: Single cell RNA-sequencing
Bulk-RNA-seq: Bulk-RNA-sequencing
AI: Artificial intelligence
OS: Overall survival
Neo-chemotherapy: Neoadjuvant chemotherapy
CA125: Carbohydrate antigen 125
CA19-9: Carbohydrate antigen 19-9
SCCA: Squamous cell carcinoma antigen
NEUT%: Neutrophil percentage
LY%: Lymphocyte percentage
MO%: monocyte percentage
NEUT#: Neutrophil count
PLT#: Platelet count NLR, neutrophil lymphocyte ratio
NPR: neutrophil platelet ratio
SII: Systemic immune-inflammation index
SIRI: Systemic inflammatory response Index
PIV: Pan-immune-inflammation value
ROC: Receiver operating characteristic
UMIs: Unique molecular identifiers
UMAP: Uniform Manifold Approximation and Projection
DEGs: Differentially expressed genes
ssGSEA: Single sample gene set enrichment analysis
VIF: Variance Inflation Factor
RFECV: Feature elimination with cross‐validation
LR: Logistic regression
RF: Random forest
NB: Naive bayes
DT: Decision tree
NNET: Neural network
AUC: The area under the receiver operating characteristic curve
NRI: Net Reclassification Improvement
IDI: Integrated Discrimination Improvement
DCA: Decision curve analysis
ORs: Odd ratios
CIs: Confidence intervals
HR: Hazard Ratio
TAMs: Tumor-associated macrophages.
