# Supplementary material for: Predicting preoperative lymph node status in patients with cervical cancer: development of interpretable machine learning model and support for the biological plausibility
Source: Front Immunol. 2025 Oct 10;16:1654332. doi: 10.3389/fimmu.2025.1654332 (PMC12549587; doi:10.3389/fimmu.2025.1654332)
Supplement: Supplementary file 1 [file Table1.docx]

**Supplementary Materials**

**Supplementary Figure 1:** Flowchart of patients’ selection.

**Supplementary Figure 2:** **The results of RFECV for 5 ML models.** Each plot shows the correlation between the number of variables and both the cross-validation accuracy. **(A):** NB, **(B):** DT, **(C):** RF, **(D):** NNET, **(E):** LR.

**Supplementary Figure 3:** **The feature importance ranking obtained through RFECV.** **(A):** NB, **(B):** DT, **(C):** RF, **(D):** NNET, **(E):** LR.

**Supplementary Figure 4:** **DCA curves for each model in train set (A), retrospective test set (B) and prospective test set (C).**

**Supplementary Figure 5:** **(A–E)** are the SHAP-related graphs of the key variables. The impact of the top 5 variables and the variables most relevant to their respective interactions on SHAP values, with each point representing a single patient in the dataset.

**Supplementary Table 1: Explore the potential predictive value of monocytes.
Supplementary Table 2: VIF for all variables.**

**Supplementary Table 3: Summary table of methods for five ML models.**

**Supplementary Table 4: Brier scores for five ML models.**

**Supplementary Table 5: The performance of the five ML models.**

**Supplementary Table 6: Delong test results of AUC differences between five ML models.**

**Supplementary Table 7: The NRI and IDI comparison of five ML models.**


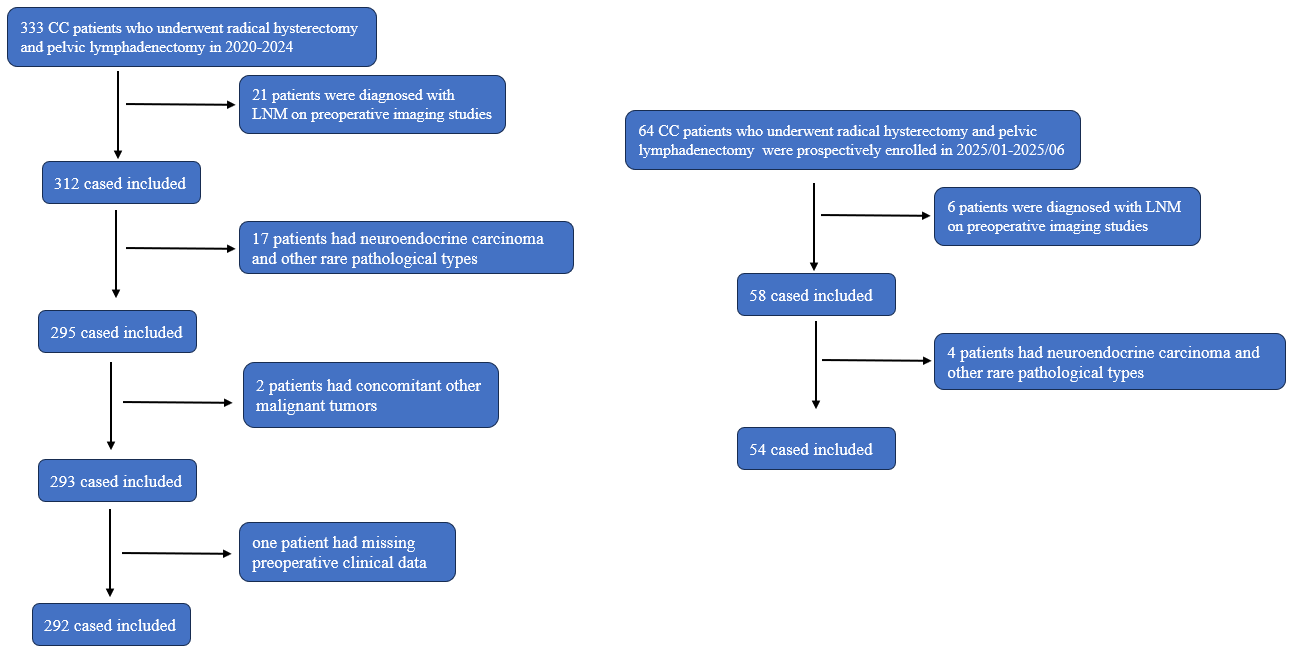


**Supplementary Figure 1:** Flowchart of patients’ selection.


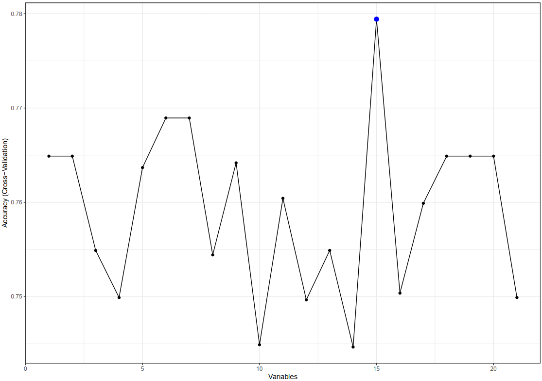

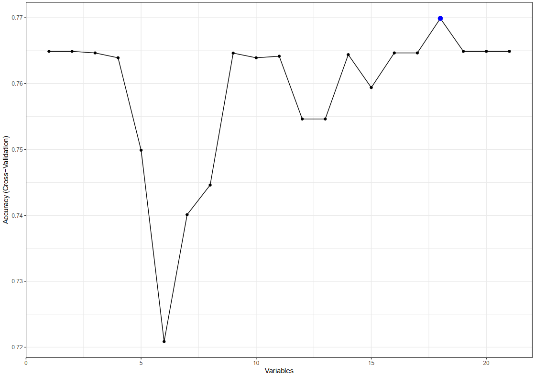


**A B**


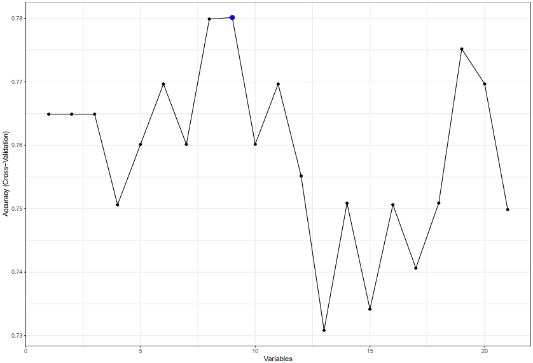

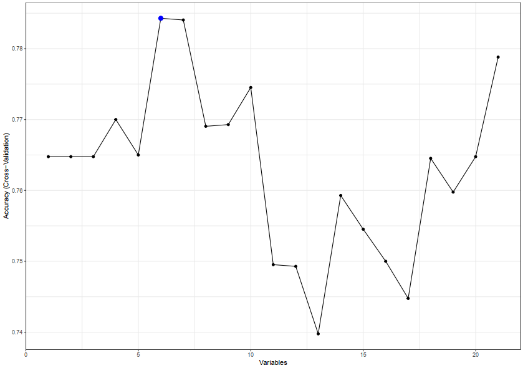


**C D**


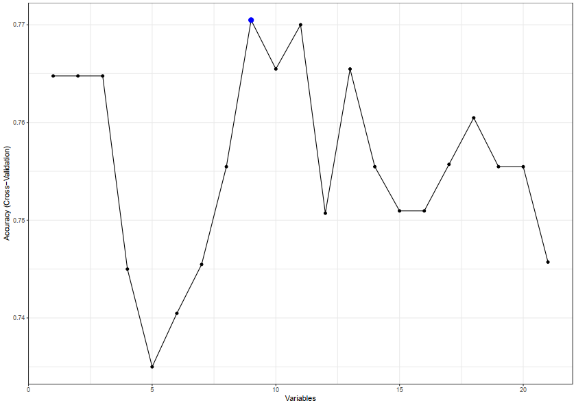


**E**

**Supplementary Figure 2:** **The results of RFECV for 5 ML models.** Each plot shows the correlation between the number of variables and both the cross-validation accuracy. **(A):** NB, **(B):** DT, **(C):** RF, **(D):** NNET, **(E):** LR.


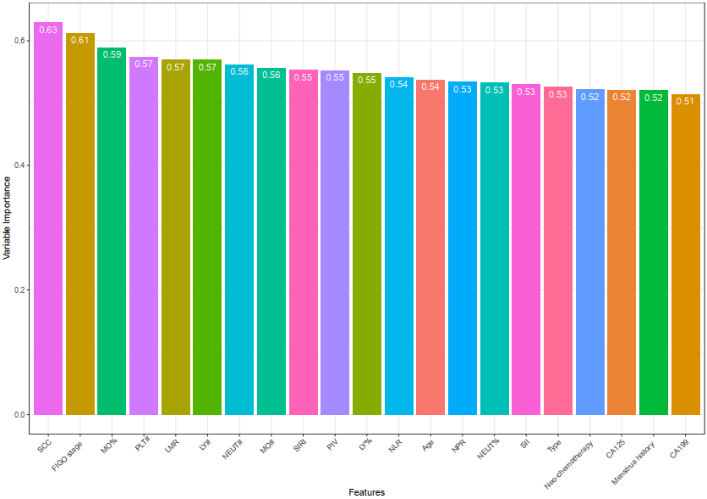

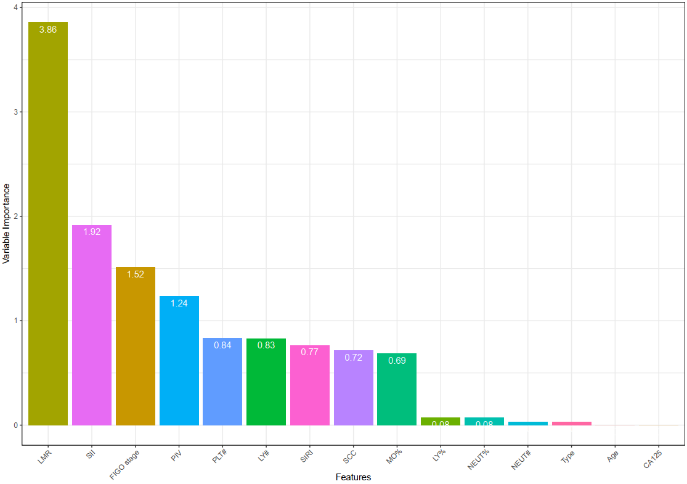


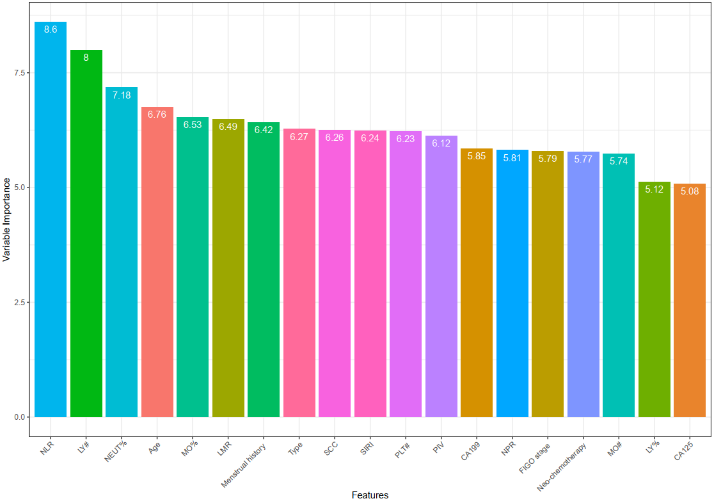
  **A B**


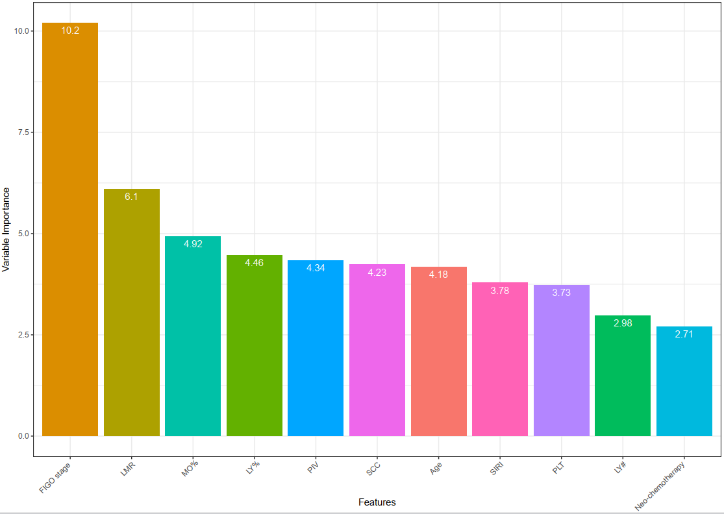


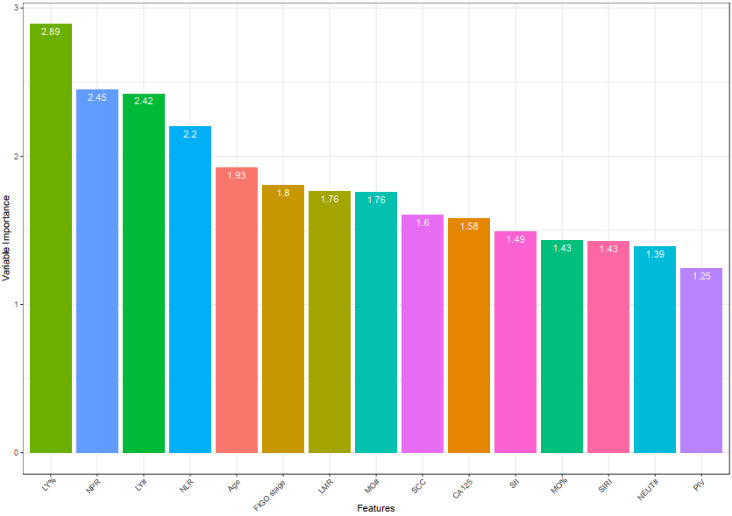
 **C D**

**E**

**Supplementary Figure 3:** **The feature importance ranking obtained through RFECV.** **(A):** NB, **(B):** DT, **(C):** RF, **(D):** NNET, **(E):** LR.


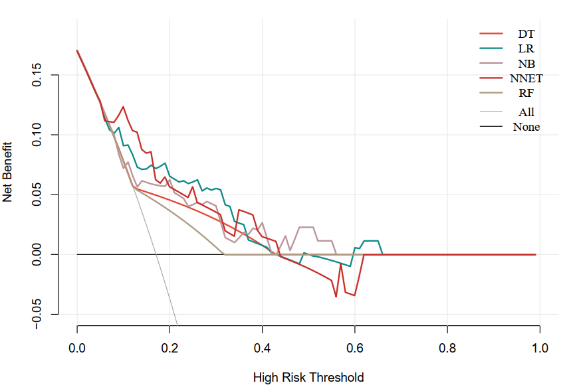

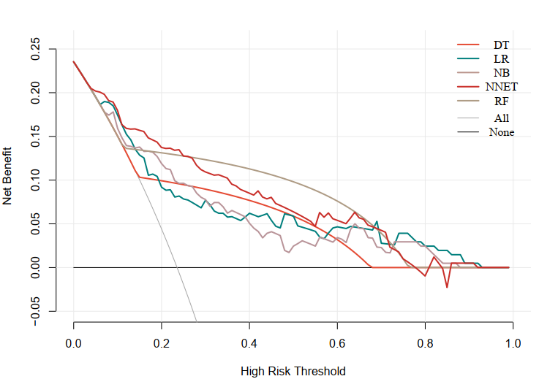


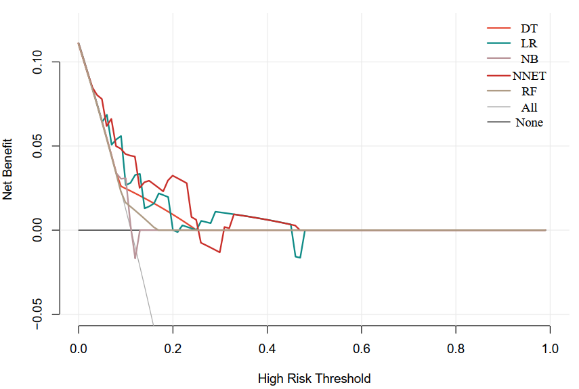
 **A B**

**C**

**Supplementary Figure 4:** **DCA curves for each model in train set (A), retrospective test set (B) and prospective test set (C).**


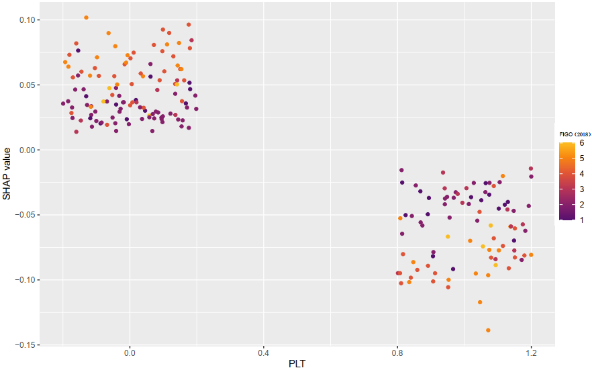

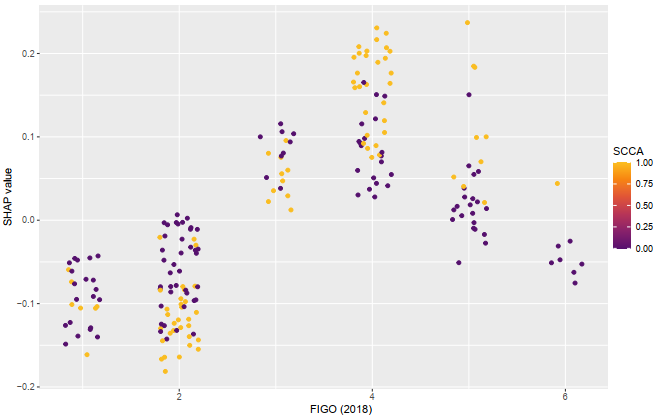


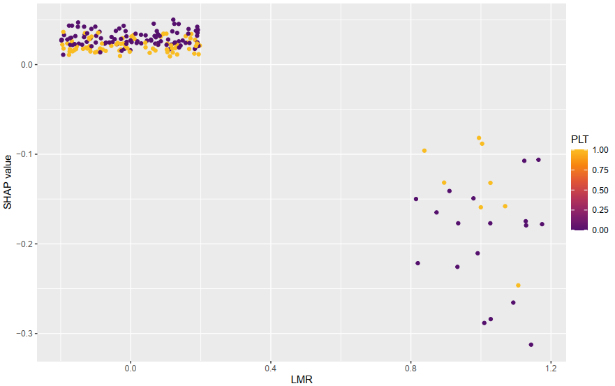

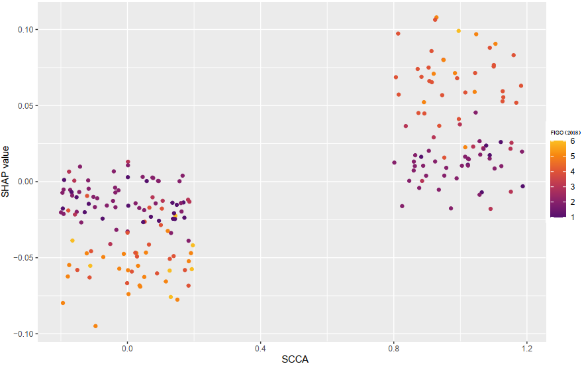
 **A B**


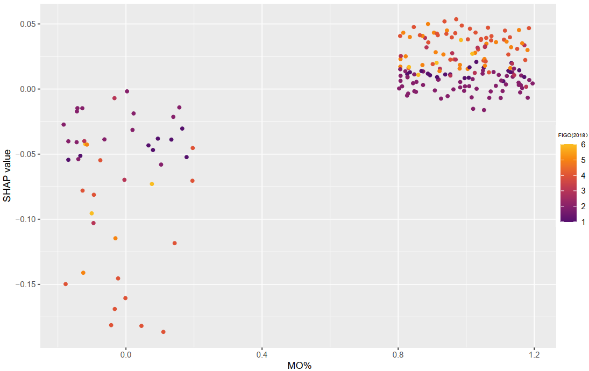
 C D

E
**Supplementary Figure 5:** **(A–E)** are the SHAP-related graphs of the key variables. The impact of the top 5 variables and the variables most relevant to their respective interactions on SHAP values, with each point representing a single patient in the dataset.

| **Model** | **AUC (95% CI)** | **Delong test** |
| --- | --- | --- |
| **Model1 (FIGO+SCCA+PLT#)** | 0.68(0.62-0.74) | *p*<0.05 |
| **Model2 (FIGO+SCCA+PLT#+MO#)** | 0.74(0.67-0.80) |  |

**Supplementary Table 1: Explore the potential predictive value of monocytes.**

**Supplementary Table 2: VIF for all variables.**

| **Variables** | **VIF** |
| --- | --- |
| **Age** | 1.33 |
| **Menstrual history** | 1.34 |
| **Neo-treatment** | 1.87 |
| **FIGO (2018)** | 1.6 |
| **Type** | 1.08 |
| **CA125** | 1.15 |
| **CA199** | 1.07 |
| **SCCA** | 1.5 |
| **NEUT%** | 3.08 |
| **LY%** | 3.62 |
| **MO%** | 1.78 |
| **NEUT#** | 2.89 |
| **LY#** | 1.88 |
| **MO#** | 2.04 |
| **PLT** | 1.86 |
| **NLR** | 2.7 |
| **LMR** | 1.93 |
| **NPR** | 2.18 |
| **SIRI** | 2.83 |
| **SII** | 3.09 |
| **PIV** | 1.89 |

| Model | variables number（RFECV） | The name of variables（RFECV） | Hyperparameter |
| --- | --- | --- | --- |
| NB | **18** | **"SCCA”,"FIGO (2018)”,"MO%","PLT","LY#","LMR”,"NEUT#","MO#","SIRI”,"PIV", "LY%”,"NLR”, "Age", "NEUT%","NPR", "SII", "Type”, "Menstrual history"** | **usekernel = TRUE, laplace = 0, adjust =1** |
| DT | **15** | **"PLT","FIGO (2018)","LY#",**  **"LMR","MO%","SII","SCCA","PIV","SIRI","LY%","NEUT%","NEUT#","Type","Age","CA125"** | **cp = 0** |
| RF | **6** | **"FIGO (2018)", "LMR", "MO%", "SCCA", "Age"，"SIRI"** | **mtry =4, min.node.size=1, splitrule="gini"** |
| NNET | **9** | **"LY#", "MO%", "Age", "NEUT%", "SCCA”, "PLT", "Menstrual history", "LMR","FIGO (2018)"** | **size = 3, decay = 0.1** |
| LR | **9** | **"LY%", "NPR", "LY#", "NLR", "Age", "FIGO (2018)","MO#","LMR","SCCA"** | **none** |

**Supplementary Table 3: Summary table of methods for five ML models.**

| Model | Brier (Train Set) | Brier (Retrospective Test Set) | Brier (Prospective Test Set) |
| --- | --- | --- | --- |
| NB | 0.14 | 0.14 | 0.14 |
| DT | 0.18 | 0.19 | 0.19 |
| RF | 0.14 | 0.26 | 0.26 |
| NNET | 0.12 | 0.13 | 0.11 |
| LR | 0.14 | 0.14 | 0.12 |

**Supplementary Table 4: Brier scores for five ML models.**

| Train Set | | | | | | |
| --- | --- | --- | --- | --- | --- | --- |
| Model | AUC (95% CI) | Sensitivity (95% CI) | Specificity (95% CI) | Accuracy (95% CI) | Youden Index (95% CI) | Best threshold (95% CI) |
| NB | 0.79(0.72-0.86) | 0.77(0.65-0.89) | 0.71(0.63-0.78) | 0.72(0.66-0.78) | 0.48(0.39-0.62) | 0.19(0.16-0.39) |
| DT | 0.70(0.63-0.78) | 0.48(0.34-0.62) | 0.93(0.89-0.97) | 0.82(0.77-0.88) | 0.41(0.26-0.54) | 1.00(1.00-1.00) |
| RF | 0.77(0.70-0.85) | 0.60(0.47-0.74) | 0.94(0.91-0.98) | 0.83(0.79-0.89) | 0.55(0.42-0.68) | 1.00(1.00-1.00) |
| NNET | 0.86(0.81-0.92) | 0.79(0.68-0.91) | 0.79(0.72-0.85) | 0.79(0.73-0.85) | 0.58(0.48-0.73) | 0.23(0.15-0.37) |
| LR | 0.79(0.72-0.86) | 0.71(0.58-0.84) | 0.71(0.63-0.78) | 0.71(0.64-0.77) | 0.39(0.36-0.57) | 0.15(0.09-0.50) |
| Retrospective Test Set | | | | | | |
| Model | AUC (95% CI) | Sensitivity (95% CI) | Specificity (95% CI) | Accuracy (95% CI) | Youden Index (95% CI) | Best threshold (95% CI) |
| NB | 0.70(0.53-0.86) | 0.60(0.35-0.85) | 0.81(0.71-0.90) | 0.77(0.69-0.86) | 0.41(0.23-0.70) | 0.33(0.10-0.61) |
| DT | 0.65(0.51-0.78) | 0.40(0.15-0.65) | 0.89(0.82-0.96) | 0.81(0.72-0.89) | 0.29(0.04-0.54) | 1.00(1.00-1.00) |
| RF | 0.63(0.49-0.77) | 0.47(0.21-0.72) | 0.79(0.70-0.89) | 0.78(0.70-0.87) | 0.26(0.00-0.50) | 1.00(1.00-1.00) |
| NNET | 0.79(0.67-0.92) | 0.93(0.81-1.00) | 0.66(0.55-0.77) | 0.70(0.61-0.80) | 0.59(0.43-0.74) | 0.20(0.20-0.40) |
| LR | 0.76(0.61-0.91) | 0.67(0.43-0.91) | 0.82(0.73-0.91) | 0.80(0.71-0.88) | 0.49(0.32-0.74) | 0.37(0.15-0.57) |
| Prospective Test Set | | | | | | |
| Model | AUC (95% CI) | Sensitivity (95% CI) | Specificity (95% CI) | Accuracy (95% CI) | Youden Index (95% CI) | Best threshold (95% CI) |
| NB | 0.52(0.28-0.77) | 0.83(0.54-1.00) | 0.46(0.32-0.60) | 0.50(0.37-0.63) | 0.29(0.08-0.60) | 0.12(0.06-0.31) |
| DT | 0.60(0.39-0.82) | 0.33(0.04-0.71) | 0.88(0.78-0.97) | 0.81(0.71-0.92) | 0.21(0.00-0.58) | 1.00(1.00-1.00) |
| RF | 0.56(0.35-0.78) | 0.33(0.04-0.71) | 0.79(0.68-0.91) | 0.74(0.62-0.86) | 0.12(0.00-0.79) | 1.00(1.00-1.00) |
| NNET | 0.76(0.54-0.98) | 0.83(0.54-1.00) | 0.69(0.56-0.82) | 0.70(0.58-0.83) | 0.52(0.25-0.87) | 0.22(0.13-0.57) |
| LR | 0.71(0.49- 0.93) | 0.83(0.54-1.00) | 0.63(0.49-0.76) | 0.65(0.52-0.78) | 0.46(0.24-0.73) | 0.20(0.05-0.72) |

**Supplementary Table 5: The performance of the five ML models.**

| Train set | | |
| --- | --- | --- |
| New model vs. Baseline model | **ΔAUC** | **DeLong's test *P* value** |
| NB vs. DT (0.79 vs. 0.70) | 0.09 | 0.06 |
| NB vs. RF (0.79 vs. 0.77) | 0.02 | 0.72 |
| NB vs. NNET (0.79 vs. 0.86) | -0.07 | 0.02 |
| NB vs. LR (0.79 vs. 0.79) | 0.00 | 0.95 |
| DT vs. RF (0.70 vs. 0.77) | -0.07 | 0.04 |
| DT vs. NNET (0.70 vs. 0.86) | -0.16 | <0.01 |
| DT vs. LR (0.70 vs. 0.79) | -0.09 | 0.76 |
| RF vs. NNET (0.77 vs. 0.86) | -0.09 | <0.01 |
| RF vs. LR (0.77 vs. 0.79) | -0.02 | 0.66 |
| NNET vs.LR (0.86 vs. 0.79) | 0.07 | 0.03 |
| Retrospective Test Set | | |
| New model vs. Baseline model | **ΔAUC** | **DeLong's test *P* value** |
| NB vs. DT (0.70 vs. 0.65) | 0.05 | 0.57 |
| NB vs. RF (0.70 vs. 0.63) | 0.07 | 0.3 |
| NB vs. NNET (0.70 vs. 0.79) | -0.09 | 0.14 |
| NB vs. LR (0.70 vs. 0.76) | -0.06 | 0.28 |
| DT vs. RF (0.65 vs. 0.63) | 0.02 | 0.82 |
| DT vs. NNET (0.65 vs. 0.79) | -0.14 | 0.02 |
| DT vs. LR (0.65 vs. 0.76) | -0.11 | 0.23 |
| RF vs. NNET (0.63 vs. 0.79) | -0.16 | 0.05 |
| RF vs. LR (0.63 vs. 0.76) | -0.13 | 0.01 |
| NNET vs.LR (0.79 vs. 0.76) | 0.03 | 0.71 |
| Prospective Test Set | | |
| New model vs. Baseline model | **ΔAUC** | **DeLong's test *P* value** |
| NB vs. DT (0.52 vs.0.60) | -0.08 | 0.49 |
| NB vs. RF (0.52 vs. 0.56) | -0.04 | 0.83 |
| NB vs. NNET (0.52 vs. 0.76) | -0.24 | <0.01 |
| NB vs. LR (0.52 vs. 0.71) | -0.19 | 0.01 |
| DT vs. RF (0.60 vs. 0.56) | 0.04 | 0.82 |
| DT vs. NNET (0.60 vs. 0.76) | -0.16 | 0.13 |
| DT vs. LR (0.60 vs. 0.71) | -0.11 | 0.40 |
| RF vs. NNET (0.56 vs. 0.76) | -0.20 | 0.28 |
| RF vs. LR (0.56 vs. 0.71) | -0.15 | 0.40 |
| NNET vs.LR (0.76 vs. 0.71) | 0.05 | 0.51 |

**Supplementary Table 6: Delong test results of AUC differences between five ML models.**

**Supplementary Table 7: The NRI and IDI comparison of five ML models.**

| Train set | | | | |
| --- | --- | --- | --- | --- |
| New model vs. Baseline model | **NRI** | ***P* value** | **IDI** | ***P* value** |
| NNET vs. NB | 0.10[-0.08-0.26] | 0.03 | 0.12[0.04-0.20] | <0.01 |
| NNET vs. DT | 0.17[0.02-0.32] | 0.60 | 0.13[0.05-0.20] | <0.01 |
| NNET vs. RF | 0.03[-0.1-0.17] | 0.23 | -0.01[-0.10-0.07] | 0.78 |
| NNET vs.LR | 0.07[-0.07-0.21] | 0.30 | 0.11[0.01-0.21] | 0.03 |
| Retrospective Test Set | | | | |
| New model vs. Baseline model | **NRI** | ***P* value** | **IDI** | ***P* value** |
| NNET vs. NB | 0.18[-0.06-0.48] | 0.82 | 0.04[-0.06-0.14] | 0.47 |
| NNET vs. DT | 0.30[0.03-0.59] | 0.42 | 0.06[0.00-0.13] | 0.06 |
| NNET vs. RF | 0.33[0.05-0.60] | 0.04 | 0.10[0.03-0.17] | <0.01 |
| NNET vs.LR | 0.10[-0.18-0.41] | 0.21 | 0.05 [-0.03-0.13] | 0.24 |
| Prospective Test Set | | | | |
| New model vs. Baseline model | **NRI** | ***P* value** | **IDI** | ***P* value** |
| NNET vs. NB | 0.23[0.04-0.41] | 0.28 | 0.10[-0.03-0.23] | 0.13 |
| NNET vs. DT | 0.31[-0.17-0.81] | 0.02 | 0.07[-0.02-0.16] | 0.12 |
| NNET vs. RF | 0.40[-0.36-0.94] | 0.07 | 0.09[-0.04-0.23] | 0.17 |
| NNET vs.LR | 0.06[-0.10-0.25] | 0.68 | 0.03[-0.03-0.09] | 0.29 |
